# SGLT2 Inhibitors and GLP-1 Receptor Agonists in Cardiovascular–Kidney–Metabolic Syndrome

**DOI:** 10.3390/biomedicines13081924

**Published:** 2025-08-07

**Authors:** Aryan Gajjar, Arvind Kumar Raju, Amani Gajjar, Mythili Menon, Syed Asfand Yar Shah, Sourbha Dani, Andrew Weinberg

**Affiliations:** 1 Department of Internal Medicine, Division of Cardiology, David Geffen School of Medicine at UCLA, Los Angeles, CA 90095, USA; 2 Department of Cardiology, Mayo Clinic Institute, Rochester, MN 55905, USA; 3 Department of Medicine, Saint Vincent Hospital, Worcester, MA 01608, USA; 4 Division of Cardiovascular Medicine, Department of Medicine, Lahey Hospital and Medical Center, Burlington, MA 01805, USA; 5 Department of Cardiology, SUNY Upstate Medical University, Syracuse, NY 13210, USA; weinbean@upstate.edu

**Keywords:** cardiovascular–kidney–metabolic (CKM) syndrome, SGLT2 inhibitors, GLP-1 receptor agonists, cardiorenal protection, type 2 diabetes mellitus

## Abstract

Cardiovascular–Kidney–Metabolic (CKM) syndrome symbolizes a single pathophysiologic entity including obesity, type 2 diabetes, chronic kidney disease, and cardiovascular disease. These conditions altogether accelerate adverse outcomes when they coexist. Recent evidence has shown that the function of glucagon-like peptide-1 receptor agonists (GLP-1RA) and sodium–glucose cotransporter-2 inhibitors (SGLT2i) alleviate stress on multiple organs. SGLT2i has been demonstrated to benefit heart failure, hemodynamic regulation, and renal protection while GLP-1RA on the other hand has been shown to demonstrate a strong impact on glycemic management, weight loss, and atherosclerotic cardiovascular disease. This review will aim to understand and evaluate the mechanistic rationalization, clinical evidence, and the potential therapeutic treatment of SGLT2 inhibitors and GLP-1 receptor agonists to treat individuals who have CKM syndrome. This analysis also assesses whether combination therapy can be a synergistic approach that may benefit patients but is still underutilized because of the lack of clear guidelines, the associated costs, and disparities in accessibility. Therefore, in this review, we will be discussing the combination therapy’s additive and synergistic effects, current recommendations and clinical evidence, and mechanistic insights of these GLT2 inhibitors and GLP-1 receptor agonists in CKM syndrome patients. Overall, early and combination usage of GLP-1RA and SGLT2i may be essential to demonstrating a significant shift in modern cardiometabolic therapy toward patient-centered care.

## 1. Introduction

Cardiovascular–Kidney–Metabolic (CKM) syndrome refers to the complex and interdependent relationship among cardiovascular disease (CVD), chronic kidney disease (CKD), diabetes mellitus, and obesity, which together form a unified pathophysiological entity. It is characterized by a cascading effect in which dysfunction in one organ system accelerates deterioration in others, amplifying disease progression and worsening clinical outcomes. Unlike isolated conditions that may independently affect multiple systems, CKM syndrome is defined by its synergistic pathology, where the combined burden is multiplicative rather than additive, leading to accelerated disease progression and worse clinical outcomes [1].

Approximately 40% of individuals with type 2 diabetes develop chronic kidney disease, and individuals with chronic kidney disease are two to four times more likely to die from cardiovascular complications than progress to kidney failure [2]. This complex network between cardiovascular disease, chronic kidney disease, and type 2 diabetes mellitus (T2DM) is driven by chronic inflammation, insulin resistance, endothelial dysfunction, hemodynamic stress, and neurohormonal activation which allows for widespread organ dysfunction. Common risk factors contributing to CKM syndrome include hypertension, excess adiposity, hyperglycemia, and elevated levels of cholesterol and triglycerides. In addition, sedentary lifestyle, poor dietary habits, and tobacco use also play a vital role in contributing to CKM syndrome. According to CDC estimates, 47% of U.S. adults have hypertension, 38% have dyslipidemia, and nearly one in two adults have prediabetes or diabetes, underscoring the widespread nature of CKM-related risk factors. Common complications of CKM syndrome include end-stage renal disease, increasing albuminuria, atherosclerotic cardiovascular disease, stroke, and decreasing glomerular filtration rate (GFR) which may lead to elevated risk of cardiovascular death, all-cause mortality, and hospitalization. Clinical studies have demonstrated that the coexistence of any two CKM-related conditions—such as diabetes and chronic kidney disease—substantially increases the risk of adverse cardiovascular outcomes, including heart failure, stroke, and cardiovascular mortality, in an exponential manner compared to the presence of a single condition [3]. For instance, individuals with both diabetes and CKD have a 3.5-fold increased risk of cardiovascular mortality compared to those with diabetes alone. The synergistic risk profile associated with the coexistence of diabetes, chronic kidney disease (CKD), and cardiovascular disease (CVD) results in disproportionately elevated rates of mortality and hospitalization compared to each condition in isolation. Historically, traditional treatment paradigms have approached CVD, CKD, and type 2 diabetes mellitus (T2DM) as distinct entities, often leading to fragmented care, overlapping pharmacologic side effects, and missed opportunities for integrated management. A systems-based holistic approach to simultaneously target the cardiovascular, renal, and metabolic systems has been shown to reduce hospitalizations, sustain organ function, and improve long-term outcomes [4,5,6].

SGLT2 inhibitors and GLP-1 receptor agonists represent paradigm-shifting drug classes that offer multi-system benefits. These agents transcend traditional organ-specific treatment boundaries by simultaneously improving glycemic control, providing renoprotective effects, and reducing the risk of major cardiovascular events, thereby exemplifying a more integrated approach to managing CKM syndrome. SGLT2i presents pleiotropic effects beyond lowering glucose levels, including reducing heart failure and slowing down the progression of CKD while GLP-1RA works to reduce atherosclerotic CV events and preserve renal function. Thus, their combined use targets all three domains of CKM, that is, the heart, kidney, and metabolic disease [7]. Despite robust clinical evidence, implementation remains inconsistent due to gaps in provider awareness, fragmented specialty care, and disparities in access. Low prescribing rates, therapeutic inertia, and limitations in access all play a role in affecting the implementation of the agents [8].

**Figure 1:**
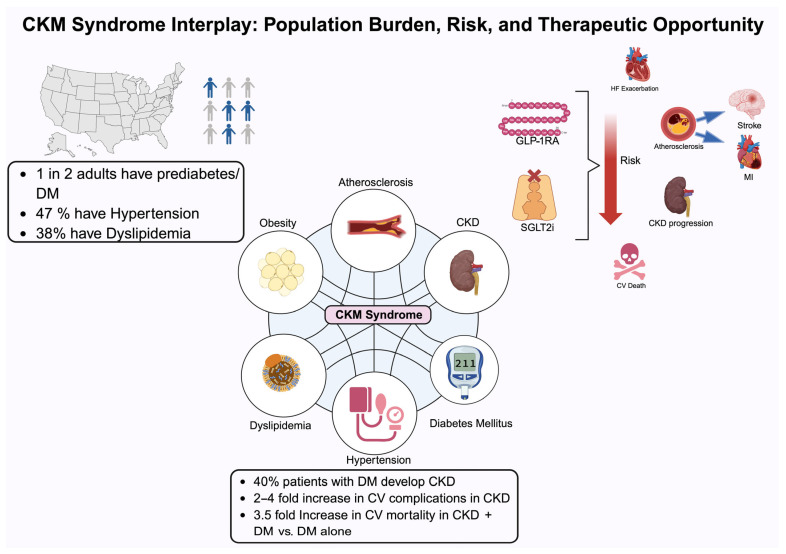
This figure illustrates the interrelated components of cardiovascular–kidney–metabolic (CKM) syndrome and highlights the substantial population-level burden, elevated risk of complications, and potential therapeutic opportunities. Abbreviations: CKD, chronic kidney disease; CV, cardiovascular; DM, type 2 diabetes mellitus; GLP-1RA, glucagon-like peptide 1 receptor agonist; HF, heart failure; MI, myocardial infarction; SGLT2i, sodium–glucose cotransporter 2 inhibitor.

This review summarizes clinical trial data and real-world studies supporting the holistic benefits of SGLT2 inhibitors and GLP-1 receptor agonists in patients with CKM syndrome (Figure 1).

## 2. Mechanistic Insights

The pathophysiology of CKM syndrome reflects a complex interplay between metabolic dysregulation, cardiovascular strain, and progressive kidney dysfunction. 

T2DM and obesity are chronic inflammatory disease processes which affect multi organ systems especially cardiac, kidney, and the vascular system. Macrophages have a pivotal role in the disease process, by both initiating and maintaining an inflammatory state [9,10,11,12,13]. When a monocyte enters a tissue, they are differentiated into either M1 macrophage which is pro-inflammatory or M2 macrophage which is anti-inflammatory depending on the cytokine milieu. Hyperglycemia in itself promotes pro-inflammatory cytokines, which leads to increased differentiation to M1 macrophages [14,15,16,17]. As a consequence, insulin resistance develops, further disrupting glucose homeostasis leading to a vicious cycle of further inflammation [18,19]. There is reduction in pro-inflammatory markers like hsCRP, IL-6, IL-1β, TNF-α, and IFN-γ in patients with diabetes and in vitro models without diabetes, while there is a change in polarity from M1 to M2 macrophages when treated with SGLT2i [11,20,21,22,23,24,25]. Similar anti-inflammatory effects are shown in in vitro studies with GLP-1RA in both diabetic and non-diabetic models, which suppress NF-κB activation and the expression of various inflammatory factors, including vascular cell adhesion molecule-1 (VCAM-1), ICAM-1, and endothelin 1 [26,27,28].

SGLT2 inhibitors reduce glucose and sodium reabsorption which leads to an increased urinary excretion of both glucose and sodium. This leads to osmotic diuresis and natriuresis which causes a reduction in plasma volume and blood pressure [29]. This causes a reduction in cardiac preload and afterload which leads to a reduction in the stress at the myocardial wall which helps in improving cardiac function—especially important for heart failure patients with preserved or decreased ejection fraction. Therefore, it helps in preserving intravascular volume while having a limited effect on imbalances in electrolytes [30].

Metabolically, SGLT2 leads to a change in the utilization of cardiac energy substrates which help in increasing β-hydroxybutyrate circulation. These ketones serve as an energy source to the body and play an important role by decreasing oxidative stress and increasing cardiac energy efficiency leading to increased cardiac function during conditions such as heart failure. In terms of the renal effects as these agents play a role in the reduction of glucose and increased reabsorption, it leads to the delivery of sodium to the macula densa and the tubuloglomerular feedback mechanisms’ activation. In addition, there is reduction in the intraglomerular pressure, which prevents the hyperfiltration that is frequently seen in the early stages of diabetic nephropathy. Furthermore, SGLT2 inhibitors also lead to mild natriuresis and osmotic diuresis causing a decrease in systemic blood pressure and plasma volume which leads to prolonging the reduction of renal hemodynamic stress. They also provide an important role in increasing the consumption and synthesis of ketone bodies leading to decreased oxidative stress in renal tubular cells and improves mitochondrial efficiency. Leading contributors to CKD such as inflammation, fibrosis, and hypoxia are reduced by these metabolic adaptations. Clinical trials such as CREDENCE and DAPA-CKD have demonstrated a reduction in albumin leading to reduced prevalence of end-stage kidney disease [31]. Moreover, they also modify pathways like NF-κB (nuclear factor-kappa B) decreasing the production of cytokines that promote inflammation. In addition, they also play an important role affecting the metabolism of uric acid, endothelial function, and sympathetic nervous system. Therefore, these mechanisms depict the importance of SGLT2 inhibition in the management of CKM syndrome [32].

GLP-1RAs are a class of medication that lowers glucose-inhibiting postprandial glucagon and therefore increases glucose-dependent insulin secretion [33]. They act on the hypothalamus by activating receptors in the arcuate nucleus, thereby regulating appetite and satiety through hypothalamic signaling pathways. The activation of GLP-1 receptors leads to the restriction of the signal of hunger causing a reduction in the intake of calories and therefore causing weight loss. Therefore, GLP-1RAs influence cardiovascular risk, lipid profiles, and insulin sensitivity [34].

GLP-1RAs play a vital role in cardiovascular protection by decreasing systemic blood pressure, enhancing endothelial function, and inhibiting the development of atherosclerotic plaque. In addition, GLP-1RAs also modulate inflammatory through lowering oxidative stress and restricting macrophage invasion. Therefore, these changes will result in major adverse cardiovascular events (MACE), particularly for those who have formed atherosclerosis and type 2 diabetes. In addition to the cardiovascular benefits of GLP-1RAs, they also provide several renal benefits [35]. One such benefit includes a reduction in albuminuria which leads to an improvement in renal hemodynamics. In addition to these benefits, specific GLP-1RAs such as semaglutide and liraglutide play a role in eGFR reduction in high-risk individuals [36].

**Figure 2:**
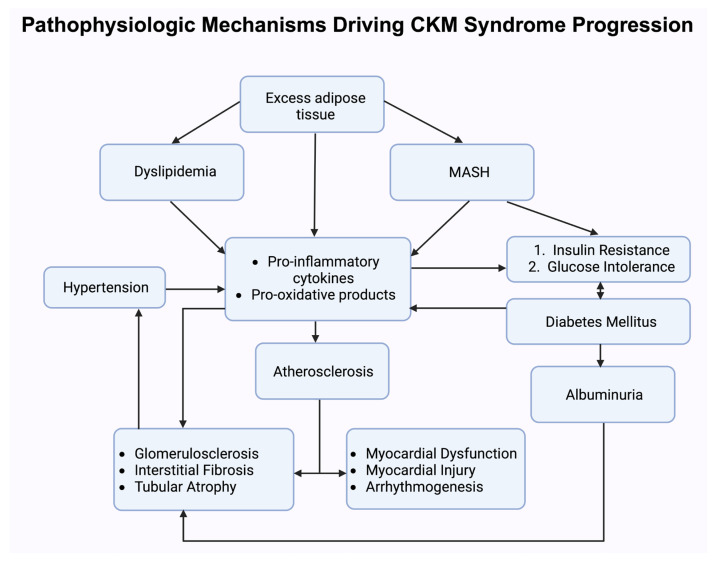
This schematic illustrates the interconnected biological pathways underlying cardiovascular–kidney–metabolic (CKM) syndrome progression. Abbreviations: MASH, metabolic dysfunction-associated steatohepatitis.

SGLT2 mainly focuses on renal hemodynamics and cardiac energy balance while GLP-1RAs have a major impact on vascular health, insulin secretion, and weight regulation. A deeper understanding of these mechanisms can help in therapies based on guidelines and the innovation of new drugs and therapies to improve patient outcomes in patients with cardiovascular kidney metabolism (Figure 2).

## 3. Clinical Evidence and Landmark Trials

### 3.1. SGLT2 Inhibitors

The EMPA-REG OUTCOME (Empagliflozin Cardiovascular Outcome Event Trial in Type 2 Diabetes Mellitus Patients) study was the first study to provide evidence that empagliflozin significantly decreased cardiovascular events. The study showed that in patients with type 2 diabetes mellitus, empagliflozin significantly reduced major adverse cardiac events (MACE) outcomes (non-fatal MI, non-fatal stroke, CV death), and it showed that unlike other interventions that reduce CV risk, like lowering blood pressure, empagliflozin showed effective results compared to a placebo very early, and a reduction in primary outcomes were evident within 3 months of starting the medication. The trial also showed a similar effect on outcome measures for both doses (10 and 25 mg) with no dose–response relationship [37].

The DAPA-HF (Dapagliflozin and Prevention of Adverse Outcomes in Heart Failure) trial provided evidence that dapagliflozin when used in patients with heart failure with reduced ejection fraction (less than 40%) significantly reduced the risk of worsening heart failure or death from cardiovascular events regardless of presence or absence of diabetes. The number of patients who would need to have been treated with dapagliflozin to prevent worsening heart failure or death from cardiovascular events was 21 [38].

The CREDENCE (Canagliflozin and Renal Events in Diabetes with Established Nephropathy Clinical Evaluation) trial predominantly studied renal outcomes, and reported that patients with type 2 diabetes and chronic kidney disease who received canagliflozin had a lower risk of the primary composite outcome of end-stage kidney disease, doubling of the serum creatinine level, or death from renal or cardiovascular causes than those who received a placebo. Patients in the canagliflozin group also had a lower risk of end-stage kidney disease, hospitalization for heart failure, and the composite of cardiovascular death, myocardial infarction, or stroke. These results were obtained on a background of the renin–angiotensin system blockade which indicates that canagliflozin may be an effective treatment option for renal and cardiovascular protection in patients with type 2 diabetes with chronic kidney disease [39].

### 3.2. GLP-1 Receptor Agonists

Similarly, the Liraglutide Effect and Action in Diabetes: Evaluation of Cardiovascular Outcome Results (LEADER) trial showed that the liraglutide group had a lower risk of first occurrence of cardiovascular death, nonfatal myocardial infarction, or nonfatal stroke in the time-to-event analysis—and lower risks of death from cardiovascular causes, death from any cause, and microvascular events than did those in the placebo group. The number of patients who would need to be treated to prevent one of the mentioned events in 3 years was 66. There was no statistically significant difference between rates of hospitalizations between the placebo and the liraglutide group [40].

**Figure 3:**
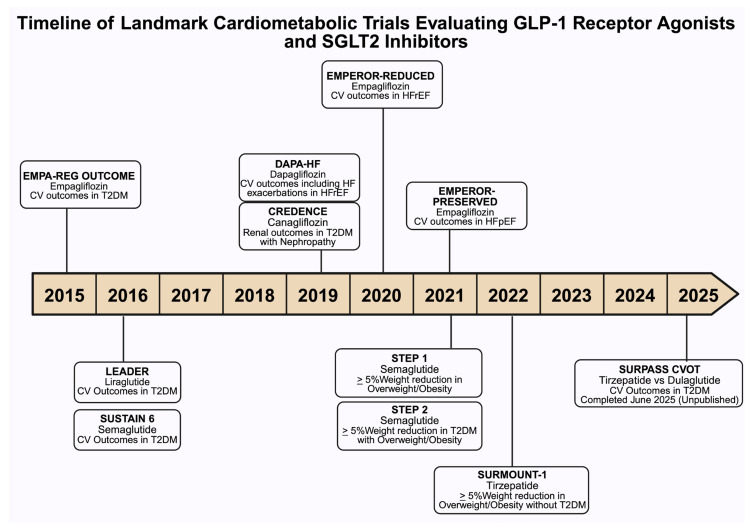
This timeline summarizes key randomized clinical trials assessing cardiovascular (CV), including heart failure (HF), renal, and metabolic outcomes associated with sodium–glucose cotransporter-2 (SGLT2) inhibitors and glucagon-like peptide-1 (GLP-1) receptor agonists in individuals with type 2 diabetes mellitus (DM), heart failure with reduced ejection fraction (HFrEF), heart failure with preserved ejection fraction (HFpEF), nephropathy, and obesity. Each entry denotes the trial name, agent studied, primary outcomes, and study population. Abbreviations: EMPA-REG OUTCOME, Empagliflozin Cardiovascular Outcomes, and mortality in DM; LEADER, Liraglutide Effect and Action in Diabetes: Evaluation of CV Outcome Results; SUSTAIN 6, Semaglutide and CV Outcomes in patients with DM; CREDENCE, Canagliflozin and renal outcomes in DM and nephropathy; DAPA-HF, Dapagliflozin in patients with HFrEF; EMPEROR-Reduced, Empagliflozin Outcomes in HFrEF; EMPEROR-Preserved, Empagliflozin Outcomes in HFpEF; STEP 1, Once-Weekly Semaglutide in Adults with Overweight or Obesity; STEP 2, Semaglutide 2·4 mg once a week in adults with overweight or obesity, and DM; SURMOUNT-1,Tirzepatide Once Weekly for the Treatment of Obesity; SURPASS CVOT, Tirzepatide compared with Dulaglutide on major CV events participants with DM.

The SUSTAIN-6 (Trial to Evaluate Cardiovascular and Other Long-term Outcomes with Semaglutide in Subjects with Type 2 Diabetes) trial evaluated the cardiovascular safety of semaglutide in patients with type 2 diabetes, demonstrating its noninferiority compared to the placebo. Semaglutide treatment resulted in a significant 26% reduction in the primary composite outcome of cardiovascular death, nonfatal myocardial infarction, or nonfatal stroke. This benefit was primarily driven by a significant 39% reduction in nonfatal stroke and a nonsignificant 26% reduction in nonfatal myocardial infarction, with no significant difference in cardiovascular mortality. Similar risk reductions were observed with both 0.5 mg and 1 mg doses of semaglutide [41] (Figure 3).

## 4. Comparative Benefits and Combination Therapy

### 4.1. SGLT2 Inhibitors

Cardiovascular disease is the most common complication of type 2 diabetes mellitus, and death due to cardiovascular disease is markedly increased in patients with diabetes. Strict glucose control reduced the effect on microvascular complications such as nephropathy and retinopathy, but for macrovascular complications, such results were not convincing [42]. Instead, strict glucose control was associated with adverse cardiovascular outcomes [43].

SGLT2 inhibitors, when used in patients with diabetes, were found to have a considerable favorable effect on cardiovascular morbidity and mortality in patients receiving the standard of care. Multiple randomized, double-blind, placebo-controlled trials showed that SGLT-2 inhibitors significantly reduced heart failure (HF) hospitalization, cardiovascular death, and all-cause mortality [37,44,45]. Additionally, SGLT2 inhibitors decreased the risk of kidney disease progression, the incidence of end-stage kidney disease, and mortality in patients with chronic kidney disease (CKD) [46,47].

### 4.2. GLP-1 Receptor Agonists

GLP-1 receptor agonists reduce hyperglycemia by glucose-dependent increases in insulin secretion and by slowing gastric emptying. Like SGLT2 inhibitors, randomized control trials of GLP-1 agonists were found to decrease cardiovascular disease events, stroke, and mortality in patients with diabetes. They also decrease new or worsening nephropathy in individuals at high risk with type 2 diabetes [41,48].

Given their ability to increase satiety, GLP-1 agonists are now one of the most demanding drugs in the United States for weight loss. The STEP-1 and STEP-2 trials showed that once-weekly semaglutide reduced body weight by 14.9% and 9.6% in non-diabetic and diabetic obese patients, respectively [40,49]. Given strong weight-reducing effects in both diabetic and non-diabetic patients without major side effects, GLP-1 receptor agonists are among the most popular drugs of the current era.

Results were even more pronounced with the dual GIP/GLP-1 agonist tirzepatide, which showed a reduction in weight up to 22.5% in the SURMOUNT-1 trial [50]. Trials to establish the efficacy of tirzepatide in reducing the risk of major adverse cardiovascular events (MACE) in people with type 2 diabetes and existing atherosclerotic cardiovascular disease (ASCVD) are still ongoing (SURPASS-CVOT); however, early findings suggest favorable outcomes. Renal outcomes and heart failure benefits remain exploratory, with ongoing trials expected to clarify its role in CKM syndrome management.

While there is strong and consistent evidence that SGLT2 inhibitors reduce heart failure hospitalization across both diabetic and non-diabetic patients with heart failure, GLP-1 receptor agonists did not show convincing benefits for heart failure patients. Major trials showed MACE reduction but no statistically significant reduction in heart failure hospitalization rates. The comparative efficacy in CKM syndrome components is summarized in Table 1.

### 4.3. Additive and Synergistic Effects of Combination Therapy

The independent effects of SGLT-2 inhibitors and GLP-1 receptor agonists on the components of CKM syndrome are described in the above section. However, the additive or synergistic effect of using them in combination is an active area of research.

The rationale behind combining the two drugs is twofold: because of their complementary mechanism of action leading to improved glycemic control and weight loss, and broader organ protection across the CKM spectrum. In other words, each agent targets different aspects of CKM syndrome. SGLT-2 inhibitors have a more pronounced renal protective effect and robust effect on heart failure hospitalization, while GLP-1 receptor agonists excel at cardiovascular protection and weight loss.

In the Duration 8 trial, patients with type 2 diabetes were randomized to receive exenatide (a GLP-1 receptor agonist), dapagliflozin (an SGLT2 inhibitor), or their combination. Combination therapy was found to have a greater reduction in weight loss, blood pressure, and HbA1c, likely due to their overlapping mechanisms [51].

Further supporting these findings, the AWARD-10 study evaluated dulaglutide (a weekly GLP-1 receptor agonist) as an add-on to SGLT-2 inhibitors in patients with suboptimal glycemic control. The combination led to improved HbA1c and body weight without increasing the risk of hypoglycemia [52]. However, both trials had relatively small sample sizes, which could be a limiting factor in generalizability.

In a more recent retrospective, observational, real-world cohort study, combination therapy with SGLT-2 inhibitors and GLP-1 receptor agonists was associated with significant reductions in major adverse cardiovascular events (MACE), heart failure hospitalizations, and progression of kidney disease, supporting the synergistic role of dual therapy in type 2 diabetes management [53].

Given their unique mechanisms of action, the SGLT-2 and GLP-1 combination seems promising for patients with CKM syndrome. Nevertheless, combination therapy needs to be tailored to each patient’s risk profile and treatment goals. Patients with considerable cardiovascular risk or kidney dysfunction, or those who have inadequate control of CKM components with monotherapy, would benefit most from the combination therapy.

### 4.4. Current Guidelines and Real-World Evidence

Current guidelines (ADA-2023, KIDGO-2022, and ESC-2023) recommend SGLT-2 inhibitors and GLP-1 receptor agonists as first-line therapies in type 2 diabetes patients with cardiovascular disease, chronic kidney disease, and heart failure. The American College of Cardiology (ACC), American Heart Association (AHA), and Heart Failure Society of America (HFSA) recommend the use of SGLT-2 inhibitors in patients with heart failure, regardless of diabetes status. SGLT-2 inhibitors are classified as Class 1 agents in patients with heart failure with reduced ejection fraction (HFrEF) and Class 2a in patients with heart failure with preserved ejection fraction (HFpEF).

Despite compelling evidence, combination therapy is not a common practice in the real world. Cost and insurance barriers are especially limiting factors for patients without adequate insurance coverage. This is exacerbated by the fact that GLP-1 receptor agonists are excessively prescribed more for weight loss rather than for their cardiovascular and metabolic benefits, leading to increased demand and limited supply. Lifestyle modification and exercise are pivotal in managing CKM syndrome.

Nevertheless, the most important factor, in our opinion, is the lack of consensus guidelines. Currently, no large, prospective, placebo-controlled cardiovascular or renal outcome trials have been specifically powered to evaluate combination therapy with SGLT-2 inhibitors and GLP-1 receptor agonists. As a result, clinicians are reluctant and raise concerns about safety profiles and adverse side effects.

In summary, SGLT-2 inhibitors and GLP-1 receptor agonists individually demonstrate robust efficacy across all the components of CKM syndrome (i.e., cardiovascular, renal, and metabolic parameters), their combination holds even greater results due to their unique complementary mechanisms and both additive and synergistic outcomes. However, real-world use is constrained by cost, accessibility, and the lack of definitive, guideline-endorsed suggestions. As we await larger, dedicated trials to validate these emerging results, the combination approach marks a new frontier in CKM management, with the potential to alter care if fully implemented in clinical practice.

## 5. Discussion

The AHA Presidential Advisory on CKM syndrome emphasizes a paradigm shift toward early identification and intervention in CKM syndrome, where SGLT2i and GLP-1RA play a crucial role.

Risk stratification involves identifying patients who would benefit from these medications using CKM staging which is based on clinical profile. In CKM stage 2, we should consider SGLT2i for CKD, and GLP-1RA for class 2 obesity or greater (BMI ≥ 35 kg/m^2^), HbA1c ≥ 9%, or high insulin requirements. A combination of SGLT2i and GLP-1RA could be utilized if multiple uncontrolled CKM risk factors are present in CKM stage 3 and it is recommended to be prioritized in stage 4 to mitigate poor cardiovascular outcomes [54]. The EMPA-REG OUTCOME trial (SGLT2i) showed a rapid onset of cardiovascular benefit, with event curve separation as early as three months, likely driven by hemodynamic effects such as volume reduction and improved heart failure outcomes [55]. In contrast, the LEADER trial (GLP-1RA) demonstrated a more gradual separation of event curves, emerging after 12–18 months, suggesting benefits primarily through slowing atherosclerotic disease progression [6]. The shift towards a shared decision-making model with multiple therapeutic options available, patient preferences regarding route of administration, side effect profiles, and cost must be integrated into treatment plans to ensure compliance. While the best treatment available is usually based on quality of evidence, translating it to the practical world is restricted by social determinants like access to resources, socioeconomic status, education, and health literacy, and efforts for equitable care remains a priority [56,57,58,59].

### 5.1. Molecular Mechanisms of Synergism

The combination of SGLT2 inhibitors and GLP-1 receptors provides beneficial therapeutic treatment therapy by focusing on different but complementary molecular pathways. For instance, when combined, the agents provide weight reduction, blood pressure reduction, and additive glycemic control. In addition, they play a vital role in the reduction of systemic glucotoxicity leading to enhanced sensitivity to insulin along with the preservation of pancreatic beta-cells. Combined treatment therapy has been shown to be more beneficial than only one agent. For instance, GLP-1RA affects an individual by reducing their appetite and delaying gastric emptying, while SGLT2i on the other hand, with the help of glucosuria, causes a reduction in calories. The synergistic combination of weight loss on the individual leads to a reduction in the visceral adiposity, a major cause of systemic inflammation and insulin resistance in CKM syndrome. Therefore, the combination of SGLT2 inhibitors and GLP-1 receptor agonists provides an effective approach for modifying the development of a medical condition and enhancing patient outcomes in high-risk groups [60,61]. 

### 5.2. Suitability, Drug Interactions, Effectiveness, and Adverse Effects of Combination Therapy

Combination therapy of SGLT2 inhibitors and GLP-1 receptor agonists is highly suitable for individuals who have CKM syndrome due to their supporting mechanisms and broad organ protection. Individuals with CKM syndrome often benefit from combination treatment therapy as they usually have intersecting renal, cardiovascular, and metabolic disorders. The combination therapy concentrates on several CKM syndrome drivers. without a substantial pharmacokinetic relationship since the two classes function through different mechanisms. However, the additive effects of the combination therapy can lead to an increase in the risk of dehydration leading to a decreased glomerular filtration rate. Adverse effects from combination therapy are usually predictable and can be managed with appropriate measures. SGLT2 inhibitors may lead to adverse effects including polyuria, vaginal infections, and volume depletion or euglycemic diabetic ketoacidosis; on the other hand, GLP-1 receptor agonists may more commonly lead to gastrointestinal symptoms including vomiting and nausea. When both are used together, in theory, it may cause fluid loss in the individual, leading to low blood pressure or inadequate oral intake, particularly in old and frail individuals. However, these effects can be minimized by adequate hydration and awareness of patients’ symptoms. Therefore, adequate monitoring of the usage of GLP-1 receptor agonists and SGLT2 inhibitors provides individuals with a safe and effective treatment of CKM syndrome [62,63,64].

### 5.3. Effectiveness, Safety and Possible Adverse Effects of Different SGLT2 Inhibitors and GLP-1 Receptor Agonists

SGLT2 inhibitor trials for empagliflozin (EMPA-REG OUTCOME), canagliflozin (CANVAS program), dapagliflozin (DECLARE-TIMI 58 and DAPA-HF), and ertugliflozin (VERTIS-CV) have demonstrated success in cardiovascular outcomes. For instance, empagliflozin and canagliflozin reduced the risk of major adverse cardiovascular events (MACE), while dapagliflozin and empagliflozin demonstrated reductions in hospitalization for heart failure. Meanwhile GLP-1 receptor agonists such as liraglutide (LEADER), semaglutide (SUSTAIN-6, PIONEER 6, SELECT), and dulaglutide (REWIND) demonstrated reduction in the risk of major adverse cardiovascular events (MACE) as well as stroke and cardiovascular risk. In addition, these agents have shown significant results in weight loss as well as playing a role in delaying the development of nephropathy. While SGLT2 inhibitors play an important role in proving cardiovascular and renal benefits, it is important to consider their safety profile. Adverse events included genital mycotic infections due to excessive amounts of glucose excretion in women. Other complications which are serious include euglycemic diabetic ketoacidosis (euDKA) in long-term fasting in patients with insulin insufficiency or minimal carbohydrate intake. In addition, older patients’ cases or those patients on concurrent diuretics faced volume depletion and hypotension, requiring monitoring of both blood pressure and hydration levels. In addition, SGLT2 inhibitors are not recommended to patients with type 1 diabetes as patients have the risk of ketoacidosis. However, the long-term effects of SGLT2 inhibitors along with newer agents are still unknown. Therefore, despite several advantages, it is necessary to weigh the potential benefits provided by the treatment against the patient’s risk profiles. It is necessary to take suitable mitigation measures including patient awareness and education, monitoring of patient’s labs, and interdisciplinary cooperation to ensure the safety of SGLT2 inhibitors [29,65,66].

Key populations remain underrepresented in the current research, including individuals with obesity without diabetes and non-diabetic patients at cardiovascular risk. Additionally, the role of GLP-1 receptor agonists in managing heart failure with preserved ejection fraction (HFpEF) among patients without diabetes or chronic kidney disease remains largely unexplored. There is also a critical need for long-term studies to determine the sustained impact of these therapies on major adverse cardiovascular events (MACE) and cardiovascular mortality. Furthermore, data on the cost effectiveness and long-term outcomes of combination therapy versus monotherapy are limited, highlighting the need for comprehensive trials to guide optimal therapeutic strategies [67].

### 5.4. Limitations and Future Perspectives

Despite this study providing a scoping review of the current literature on SGLT2 inhibitors and GLP-1 receptor agonists in Cardiovascular–Kidney–Metabolic syndrome, certain limitations should be addressed. Studies had different patient populations, endpoints, and trial durations which may lead to heterogeneity which complicates determining and comparing outcomes and efficacy. Second, the review was carried out in a narrative fashion as opposed to a systematic one. In addition, the review focused primarily on cardiovascular and renal outcomes as well as the combination treatment therapy and did not go into detail regarding inequities in access, patient adherence, and cost effectiveness. 

Future research should focus more on prospective randomized controlled trials with an emphasis on safety, efficacy, and patient outcomes of combination therapy of diverse patient populations. In addition, new combination treatment therapy should be compared with the further treatment options. As research continues towards the development of SGLT2 inhibitors and GLP-1 receptor agonists, future directions beyond diabetes are being explored. One such instance is using it for obese people with early-stage chronic kidney disease or non-diabetic people at high cardiovascular risk. There are ongoing trials such as SURPASS-CVOT and FLOW evaluating cardiovascular outcomes of tirzepatide and effects of semaglutide on kidney health in individuals with chronic kidney disease (CKD) and type 2 diabetes. In addition, the combination of specific therapies may lead to additional long-term benefits. In parallel, advancements in personalized medicines could lead to categorizations allowing for specific metabolic or genetic biomarker profiles to accurately predict specific responses towards personalized treatment therapies. The development of combination therapies which provide the mechanism of several treatment therapy methods into one single formulation reducing the number of pills is required. Long term outcome studies are necessary to validate the clinical benefits and cost effectiveness of these combination therapies. The stages of CKM syndrome, their criteria, and clinical implications are outlined in Table 2. The evolution and development of Cardiovascular–Kidney–Metabolic syndrome management will not only depend on the innovations in pharmaceuticals but also the clinical and cost effectiveness, policy reform, and cross-disciplinary collaboration with patients at the center of care.

## Figures and Tables

**Table 1:** Comparative efficacy in CKM syndrome components.

Outcome	SGLT2 Inhibitors	GLP-1 Receptor Agonists
Cardiovascular Disease (CVD)	Reduce MACE. HR 0.86 (0.74–0.99), RRR 14% (EMPA-REG OUTCOME)	Greater MACE reduction vs. SGLT2 inhibitors. HR 0.74 (0.58–0.95), RRR 26% (SUSTAIN-6)
Chronic Kidney Disease (CKD)	Reduce risk of new or worsening nephropathy in T2DM 0.61 (0.53–0.70) RRR 39% (EMPA-REG OUTCOME)	Reduce risk of new or worsening nephropathy in T2DM 0.64 (0.46–0.88) ↓ 36% (SUSTAIN-6)
HF hospitalization	Composite of CV death or HF hospitalization 0.75 (0.65–0.86), RRR ↓ 25% (EMPEROR-Reduced) HZ 0.79 (0.69–0.90), RRR ↓ 21% (EMPEROR-Preserved)	No statistically significant data to suggest efficacy in HF patients
HbA1c reduction	Modest HbA1c reduction (−0.24% to −0.58%) seen in (EMPA-REG, CANVAS, CREDENCE)	Greater HbA1c reduction (−0.4% to −1.0%) across trials (LEADER, SUSTAIN-6)
Weight loss	Minimal to modest weight loss (−1.6 kg to −2.0 kg) compared to GLP-1RAs.	Mean weight loss of −15.3 kg (or −14.9% body weight) with semaglutide (STEP 1)
Blood pressure	More pronounced BP reduction: Empagliflozin (−4.6/−1.5 mmHg, EMPA-REG OUTCOME), Canagliflozin (−3.6/−1.3 mmHg, CANVAS Program)	Modest BP reduction observed: Liraglutide (−1.6/−0.6 mmHg, LEADER), Semaglutide (−1.8/−0.5 mmHg, SUSTAIN-6)

**Table 2:** CKM syndrome stages. Abbreviations: ASCVD, atherosclerotic cardiovascular disease; BMI, body mass index; CAC, coronary artery calcium; CAD, coronary artery disease; CKD, chronic kidney disease; CKM, cardiovascular–kidney–metabolic; CT, computed tomography; CVD, cardiovascular disease; DM, diabetes mellitus; HbA1c, hemoglobin A1c; HDL, high density lipoprotein; HF, heart failure; hs-cTn, high-sensitivity cardiac troponin; HTG, hypertriglyceridemia; HTN, hypertension; MetS, metabolic syndrome; NT-proBNP, N-terminal pro-B-type natriuretic peptide; PAD, peripheral artery disease.

CKM Syndrome Staging	Definition
Stage 0: No Risk Factors	Individuals without overweight/obesity, metabolic risk factors (HTN, DM, MetS *, HTG ^#^), CKD or subclinical/clinical CVD.
Stage 1: Excess or Dysfunctional Adiposity	Individuals with Overweight/obesity—BMI ≥ 25 kg/m^2^ (or ≥23 kg/m^2^ if Asian ancestry), or Abdominal obesity—Waist circumference ≥ 88/102 cm in women/men (or if Asian ancestry ≥ 80/90 cm in women/men), or Prediabetes/impaired glucose tolerance—Blood glucose ≥ 100–124 mg/dL or HbA1c between 5.7% and 6.4% and without other metabolic risk factors or CKD
Stage 2: Metabolic Risk Factors and Kidney Disease	Individuals with metabolic risk factors and/or moderate to high-risk CKD
Stage 3: Subclinical CVD in CKM Syndrome	Individuals with Stage 1 or Stage 2 risk factors, and Subclinical ASCVD—presence of CAC identified by invasive/CT angiography, or Subclinical HF—Elevated biomarkers (NT-proBNP, hs-cTn), echocardiography findings, or Very high-risk CKD, or High predicted 10-year CVD risk
Stage 4: CVD in CKM Syndrome	Individuals with Stage 1 or Stage 2 risk factors, and clinical CVD including CAD, HF, stroke, PAD, atrial fibrillation. Stage 4a—Without kidney failure Stage 4b—With kidney failure

* MetS is defined as the presence of 3 or more of the following: (1) abdominal obesity; (2) HDL cholesterol < 40 mg/dL for men and < 50 mg/dL for women; (3) HTG; (4) elevated blood pressure (SBP ≥ 130 mmHg or DBP ≥ 80 mmHg); and (5) fasting blood glucose ≥ 100 mg/dL. # HTG is defined as triglycerides > 135 mg/dL.

## Abbreviations

The following abbreviations are used in this manuscript:

CKM	Cardiovascular–Kidney–Metabolic (Syndrome)
SGLT2i	Sodium–Glucose Cotransporter-2 Inhibitor
GLP-1RA	Glucagon-Like Peptide-1 Receptor Agonist
HFpEF	Heart Failure with Preserved Ejection Fraction

## References

[1] Sebastian S.A., Padda I., Johal G. (2024). Cardiovascular-Kidney-Metabolic (CKM) syndrome: A state-of-the-art review. Curr. Probl. Cardiol..

[2] Usman M.S., Khan M.M.S., Butler J. (2021). The Interplay Between Diabetes, Cardiovascular Disease, and Kidney Disease. Chronic Kidney Disease and Type 2 Diabetes.

[3] Swamy S., Noor S.M., Mathew R.O. (2023). Cardiovascular Disease in Diabetes and Chronic Kidney Disease. J. Clin. Med..

[4] Pagidipati N.J., Deedwania P. (2021). A Comprehensive Cardiovascular-Renal-Metabolic Risk Reduction Approach to Patients with Type 2 Diabetes Mellitus. Am. J. Med..

[5] Matsushita K., Coresh J., Sang Y., Chalmers J., Fox C., Guallar E., Jafar T., Jassal S.K., Landman G.W.D., Muntner P. (2015). Estimated glomerular filtration rate and albuminuria for prediction of cardiovascular outcomes: A collaborative meta-analysis of individual participant data. Lancet Diabetes Endocrinol..

[6] Fitchett D., Butler J., van de Borne P., Zinman B., Lachin J.M., Wanner C., Woerle H.J., Hantel S., George J.T., Johansen O.E. (2018). Effects of empagliflozin on risk for cardiovascular death and heart failure hospitalization across the spectrum of heart failure risk in the EMPA-REG OUTCOME^®^ trial. Eur. Heart J..

[7] Rolek B., Haber M., Gajewska M., Rogula S., Pietrasik A., Gąsecka A. (2023). SGLT2 Inhibitors vs. GLP-1 Agonists to Treat the Heart, the Kidneys and the Brain. J. Cardiovasc. Dev. Dis..

[8] Yau K., Dharia A., Alrowiyti I., Cherney D.Z. (2022). Prescribing SGLT2 Inhibitors in Patients With CKD: Expanding Indications and Practical Considerations. Kidney Int. Rep..

[9] Tsalamandris S., Antonopoulos A.S., Oikonomou E., Papamikroulis G.-A., Vogiatzi G., Papaioannou S., Deftereos S., Tousoulis D. (2019). The Role of Inflammation in Diabetes: Current Concepts and Future Perspectives. Eur. Cardiol. Rev..

[10] Niknejad A., Hosseini Y., Shamsnia H.S., Kashani A.S., Rostamian F., Momtaz S., Abdolghaffari A.H. (2023). Sodium Glucose Transporter-2 Inhibitors (SGLT2Is)-TLRs Axis Modulates Diabetes. Cell Biochem. Biophys..

[11] Balogh D.B., Wagner L.J., Fekete A. (2023). An Overview of the Cardioprotective Effects of Novel Antidiabetic Classes: Focus on Inflammation, Oxidative Stress, and Fibrosis. Int. J. Mol. Sci..

[12] Mantovani A., Biswas S.K., Galdiero M.R., Sica A., Locati M. (2013). Macrophage plasticity and polarization in tissue repair and remodelling. J. Pathol..

[13] Rohm T.V., Meier D.T., Olefsky J.M., Donath M.Y. (2022). Inflammation in obesity, diabetes, and related disorders. Immunity.

[14] Elmadbouh I., Singla D.K. (2021). BMP-7 Attenuates Inflammation-Induced Pyroptosis and Improves Cardiac Repair in Diabetic Cardiomyopathy. Cells.

[15] Bugger H., Abel E.D. (2014). Molecular mechanisms of diabetic cardiomyopathy. Diabetologia.

[16] Bene N.C., Alcaide P., Wortis H.H., Jaffe I.Z. (2014). Mineralocorticoid receptors in immune cells: Emerging role in cardiovascular disease. Steroids.

[17] Liu J., Zhang Y., Sheng H., Liang C., Liu H., Guerrero J.A.M., Lu Z., Mao W., Dai Z., Liu X. (2021). Hyperoside Suppresses Renal Inflammation by Regulating Macrophage Polarization in Mice With Type 2 Diabetes Mellitus. Front. Immunol..

[18] Akash M.S.H., Rehman K., Liaqat A. (2018). Tumor Necrosis Factor-Alpha: Role in Development of Insulin Resistance and Pathogenesis of Type 2 Diabetes Mellitus. J. Cell. Biochem..

[19] Fernandez-Veledo S., Vila-Bedmar R., Nieto-Vazquez I., Lorenzo M. (2009). c-Jun N-terminal kinase 1/2 activation by tumor necrosis factor-α induces insulin resistance in human visceral but not subcutaneous adipocytes: Reversal by liver X re-ceptor agonists. J. Clin. Endocrinol. Metab..

[20] Hattori S. (2018). Anti-inflammatory effects of empagliflozin in patients with type 2 diabetes and insulin resistance. Diabetol. Metab. Syndr..

[21] La Grotta R., de Candia P., Olivieri F., Matacchione G., Giuliani A., Rippo M.R., Tagliabue E., Mancino M., Rispoli F., Ferroni S. (2022). Anti-inflammatory effect of SGLT-2 inhibitors via uric acid and insulin. Cell. Mol. Life Sci..

[22] Heerspink H.J.L., Perco P., Mulder S., Leierer J., Hansen M.K., Heinzel A., Mayer G. (2019). Canagliflozin reduces inflammation and fibrosis biomarkers: A potential mechanism of action for beneficial effects of SGLT2 inhibitors in diabetic kidney disease. Diabetologia.

[23] Kim S.R., Lee S.G., Kim S.H., Kim J.H., Choi E., Cho W., Rim J.H., Hwang I., Lee C.J., Lee M. (2020). SGLT2 inhibition modulates NLRP3 inflammasome activity via ketones and insulin in diabetes with cardiovascular disease. Nat. Commun..

[24] Lee S.-G., Lee S.-J., Lee J.-J., Kim J.-S., Lee O.-H., Kim C.-K., Kim D., Lee Y.-H., Oh J., Park S. (2020). Anti-Inflammatory Effect for Atherosclerosis Progression by Sodium-Glucose Cotransporter 2 (SGLT-2) Inhibitor in a Normoglycemic Rabbit Model. Korean Circ. J..

[25] Yang H., Lan W., Liu W., Chen T., Tang Y. (2023). Dapagliflozin promotes angiogenesis in hindlimb ischemia mice by inducing M2 macrophage polarization. Front. Pharmacol..

[26] Dai Y., Mehta J.L., Chen M. (2013). Glucagon-like Peptide-1 Receptor Agonist Liraglutide Inhibits Endothelin-1 in Endothelial Cell by Repressing Nuclear Factor-Kappa B Activation. Cardiovasc. Drugs Ther..

[27] Baylan U., Korn A., Emmens R.W., Schalkwijk C.G., Niessen H.W., Krijnen P.A., Simsek S. (2022). Liraglutide treatment attenuates inflammation markers in the cardiac, cerebral and renal microvasculature in streptozotocin-induced diabetic rats. Eur. J. Clin. Investig..

[28] Zhou Y., He X., Chen Y., Huang Y., Wu L., He J. (2015). Exendin-4 attenuates cardiac hypertrophy via AMPK/mTOR signaling pathway activation. Biochem. Biophys. Res. Commun..

[29] Padda I.S., Mahtani A.U., Parmar M. (2025). Sodium-Glucose Transport Protein 2 (SGLT2) Inhibitors [Updated 3 June 2023]. StatPearls [Internet].

[30] Ni L., Yuan C., Chen G., Zhang C., Wu X. (2020). SGLT2i: Beyond the glucose-lowering effect. Cardiovasc. Diabetol..

[31] Vargas-Delgado A.P., Herrera E.A., Mite C.T., Cedeno P.D., Van Loon M.C., Badimon J.J. (2023). Renal and Cardiovascular Metabolic Impact Caused by Ketogenesis of the SGLT2 Inhibitors. Int. J. Mol. Sci..

[32] Baker R.G., Hayden M.S., Ghosh S. (2011). NF-κB, Inflammation, and Metabolic Disease. Cell Metab..

[33] Collins L., Costello R.A. (2025). Glucagon-Like Peptide-1 Receptor Agonists [Updated 29 February 2024]. StatPearls [Internet].

[34] Diz-Chaves Y., Herrera-Pérez S., González-Matías L.C., Lamas J.A., Mallo F. (2020). Glucagon-Like Peptide-1 (GLP-1) in the Integration of Neural and Endocrine Responses to Stress. Nutrients.

[35] Ma X., Liu Z., Ilyas I., Little P.J., Kamato D., Sahebka A., Chen Z., Luo S., Zheng X., Weng J. (2021). GLP-1 receptor agonists (GLP-1RAs): Cardiovascular actions and therapeutic potential. Int. J. Biol. Sci..

[36] Rroji M., Spasovski G. (2024). Transforming Diabetes Care: The Molecular Pathways through Which GLP1-RAs Impact the Kidneys in Diabetic Kidney Disease. Biomedicines.

[37] Zinman B., Wanner C., Lachin J.M., Fitchett D., Bluhmki E., Hantel S., Mattheus M., Devins T., Johansen O.E., Woerle H.J. (2015). Empagliflozin, Cardiovascular Outcomes, and Mortality in Type 2 Diabetes. N. Engl. J. Med..

[38] McMurray J.J., DeMets D.L., Inzucchi S.E., Køber L., Kosiborod M.N., Langkilde A.M., Martinez F.A., Bengtsson O., Ponikowski P., Sabatine M.S. (2019). The Dapagliflozin and Prevention of Adverse-outcomes in Heart Failure (DAPA-HF) trial: Baseline characteristics. Eur. J. Heart Fail..

[39] Perkovic V., Jardine M.J., Neal B., Bompoint S., Heerspink H.J.L., Charytan D.M., Edwards R., Agarwal R., Bakris G., Bull S. (2019). Canagliflozin and Renal Outcomes in Type 2 Diabetes and Nephropathy. N. Engl. J. Med..

[40] Marso S.P., Daniels G.H., Brown-Frandsen K., Kristensen P., Mann J.F.E., Nauck M.A., Nissen S.E., Pocock S., Poulter N.R., Ravn L.S. (2016). Liraglutide and Cardiovascular Outcomes in Type 2 Diabetes. N. Engl. J. Med..

[41] Marso S.P., Bain S.C., Consoli A., Eliaschewitz F.G., Jódar E., Leiter L.A., Lingvay I., Rosenstock J., Seufert J., Warren M.L. (2016). Semaglutide and Cardiovascular Outcomes in Patients with Type 2 Diabetes. N. Engl. J. Med..

[42] Ndumele C.E., Rangaswami J., Chow S.L., Neeland I.J., Tuttle K.R., Khan S.S., Coresh J., Mathew R.O., Baker-Smith C.M., Carnethon M.R. (2023). Cardiovascular-Kidney-Metabolic Health: A Presidential Advisory from the American Heart Association. Circulation.

[43] Beckman J.A., Creager M.A., Libby P. (2002). Diabetes and Atherosclerosis: Epidemiology, pathophysiology, and management. JAMA.

[44] Udell J.A., Cavender M.A., Bhatt D.L., Chatterjee S., Farkouh M.E., Scirica B.M. (2015). Glucose-lowering drugs or strategies and cardiovascular outcomes in patients with or at risk for type 2 diabetes: A meta-analysis of randomised controlled trials. Lancet Diabetes Endocrinol..

[45] Packer M., Anker S.D., Butler J., Filippatos G., Pocock S.J., Carson P., Januzzi J., Verma S., Tsutsui H., Brueckmann M. (2020). Cardiovascular and Renal Outcomes with Empagliflozin in Heart Failure. N. Engl. J. Med..

[46] Anker S.D., Butler J., Filippatos G., Ferreira J.P., Bocchi E., Böhm M., Brunner–La Rocca H.-P., Choi D.-J., Chopra V., Chuquiure-Valenzuela E. (2021). Empagliflozin in Heart Failure with a Preserved Ejection Fraction. N. Engl. J. Med..

[47] Heerspink H.J.L., Stefánsson B.V., Correa-Rotter R., Chertow G.M., Greene T., Hou F.-F., Mann J.F.E., McMurray J.J.V., Lindberg M., Rossing P. (2020). Dapagliflozin in Patients with Chronic Kidney Disease. N. Engl. J. Med..

[48] Herrington W.G., Staplin N., Wanner C., Green J.B., Hauske S.J., Emberson J.R., Preiss D., Judge P., Mayne K.J., The EMPA-KIDNEY Collaborative Group (2023). Empagliflozin in Patients with Chronic Kidney Disease. N. Engl. J. Med..

[49] Wilding J.P.H., Batterham R.L., Calanna S., Davies M., Van Gaal L.F., Lingvay I., McGowan B.M., Rosenstock J., Tran M.T., Wadden T.A. (2021). Once-Weekly Semaglutide in Adults with Overweight or Obesity. N. Engl. J. Med..

[50] Davies M., Færch L., Jeppesen O.K., Pakseresht A., Pedersen S.D., Perreault L., Rosenstock J., Shimomura I., Viljoen A., Wadden T.A. (2021). Semaglutide 2·4 mg once a week in adults with overweight or obesity, and type 2 diabetes (STEP 2): A randomised, double-blind, double-dummy, placebo-controlled, phase 3 trial. Lancet.

[51] Jastreboff A.M., Aronne L.J., Ahmad N.N., Wharton S., Connery L., Alves B., Kiyosue A., Zhang S., Liu B., Bunck M.C. (2022). Tirzepatide Once Weekly for the Treatment of Obesity. N. Engl. J. Med..

[52] Frías J.P., Guja C., Hardy E., Ahmed A., Dong F., Öhman P., Jabbour S.A. (2016). Exenatide once weekly plus dapagliflozin once daily versus exenatide or dapagliflozin alone in patients with type 2 diabetes inadequately controlled with metformin monotherapy (DURATION-8): A 28 week, multicentre, double-blind, phase 3, randomised controlled trial. Lancet Diabetes Endocrinol..

[53] Ludvik B., Frías J.P., Tinahones F.J., Wainstein J., Jiang H., Robertson K.E., García-Pérez L.-E., Woodward D.B., Milicevic Z. (2018). Dulaglutide as add-on therapy to SGLT2 inhibitors in patients with inadequately controlled type 2 diabetes (AWARD-10): A 24-week, randomised, double-blind, placebo-controlled trial. Lancet Diabetes Endocrinol..

[54] Chaiyakunapruk N., Tan X., Liang Y., Guevarra M., Xie L., Cheng A.Y.Y. (2025). Real-world effectiveness of adding newer generation GLP-1RA to SGLT2i in type 2 diabetes. Cardiovasc. Diabetol..

[55] Ndumele C.E., Neeland I.J., Tuttle K.R., Chow S.L., Mathew R.O., Khan S.S., Coresh J., Baker-Smith C.M., Carnethon M.R., Després J.-P. (2023). A Synopsis of the Evidence for the Science and Clinical Management of Cardiovascular-Kidney-Metabolic (CKM) Syndrome: A Scientific Statement From the American Heart Association. Circulation.

[56] Mann J.F., Fonseca V., Mosenzon O., Raz I., Goldman B., Idorn T., von Scholten B.J., Poulter N.R., LEADER Publication Committee on behalf of the LEADER Trial Investigators (2018). Effects of liraglutide versus placebo on cardiovascular events in patients with type 2 di-abetes mellitus and chronic kidney disease: Results from the LEADER trial. Circulation.

[57] Eberly L.A., Yang L., Eneanya N.D., Essien U., Julien H., Nathan A.S., Khatana S.A.M., Dayoub E.J., Fanaroff A.C., Giri J. (2021). Association of race/ethnicity, gender, and socioeconomic status with sodium-glucose cotransporter 2 inhibitor use among patients with dia-betes in the US. JAMA.

[58] McCoy R.G., Dykhoff H.J., Sangaralingham L., Ross J.S., Karaca-Mandic P., Montori V.M., Shah N.D. (2019). Adoption of New Glucose-Lowering Medications in the U.S.—The Case of SGLT2 Inhibitors: Nationwide Cohort Study. Diabetes Technol. Ther..

[59] Eberly L.A., Yang L., Essien U.R., Eneanya N.D., Julien H.M., Luo J., Nathan A.S., Khatana S.A.M., Dayoub E.J., Fanaroff A.C. (2021). Racial, ethnic, and socioeconomic inequities in glucagon-like peptide-1 receptor agonist use among patients with diabetes in the US. JAMA Health Forum.

[60] Bae J.H. (2025). SGLT2 Inhibitors and GLP-1 Receptor Agonists in Diabetic Kidney Disease: Evolving Evidence and Clinical Application. Diabetes Metab. J..

[61] Gourdy P., Darmon P., Dievart F., Halimi J.-M., Guerci B. (2023). Combining glucagon-like peptide-1 receptor agonists (GLP-1RAs) and sodium-glucose cotransporter-2 inhibitors (SGLT2is) in patients with type 2 diabetes mellitus (T2DM). Cardiovasc. Diabetol..

[62] Gao Y.-M., Feng S.-T., Wen Y., Tang T.-T., Wang B., Liu B.-C. (2022). Cardiorenal protection of SGLT2 inhibitors—Perspectives from metabolic reprogramming. eBioMedicine.

[63] Davies M.J., Drexel H., Jornayvaz F.R., Pataky Z., Seferović P.M., Wanner C. (2022). Cardiovascular outcomes trials: A paradigm shift in the current management of type 2 diabetes. Cardiovasc. Diabetol..

[64] Xie X., Wu C., Hao Y., Wang T., Yang Y., Cai P., Zhang Y., Huang J., Deng K., Yan D. (2023). Benefits and risks of drug combination therapy for diabetes mellitus and its complications: A comprehensive review. Front. Endocrinol..

[65] Kluger A.Y., Tecson K.M., Barbin C.M., Lee A.Y., Lerma E.V., Rosol Z.P., Rangaswami J., Lepor N.E., Cobble M.E., McCullough P.A. (2018). Cardiorenal Outcomes in the CANVAS, DECLARE-TIMI 58, and EMPA-REG OUTCOME Trials: A Systematic Review. Rev. Cardiovasc. Med..

[66] Husain M., Bain S.C., Jeppesen O.K., Lingvay I., Sørrig R., Treppendahl M.B., Vilsbøll T. (2020). Semaglutide (SUSTAIN and PIONEER) reduces cardiovascular events in type 2 diabetes across varying cardiovascular risk. Diabetes, Obes. Metab..

[67] Karakasis P., Fragakis N., Patoulias D., Theofilis P., Sagris M., Koufakis T., Vlachakis P.K., Rangraze I.R., El Tanani M., Tsioufis K. (2024). The Emerging Role of Glucagon-like Peptide-1 Receptor Agonists in the Management of Obesity-Related Heart Failure with Preserved Ejection Fraction: Benefits beyond What Scales Can Measure?. Biomedicines.

